# Distinct associations between gratitude, self-esteem, and optimism with subjective and psychological well-being among Japanese individuals

**DOI:** 10.1186/s40359-024-01606-y

**Published:** 2024-03-07

**Authors:** Norberto Eiji Nawa, Noriko Yamagishi

**Affiliations:** 1 Center for Information and Neural Networks (CiNet), National Institute of Information and Communications Technology (NICT), Advanced ICT Research Institute, 1-4 Yamadaoka, Suita, Osaka 565-0871 Japan; 2 Graduate School of Frontiers Biosciences, Osaka University, Suita, Osaka Japan; 3 College of Global Liberal Arts, Ritsumeikan University, Ibaraki, Osaka 567-8570 Japan

**Keywords:** Positive psychology intervention, Gratitude, Self-esteem, Optimism, Subjective well-being, Psychological well-being, Hedonia, Eudaimonia, Non-Western populations

## Abstract

**Background:**

Mounting evidence suggests that the effectiveness of positive psychology interventions is influenced by a variety of factors, including cultural context. Identifying intervention targets that can effectively contribute to improving individual well-being under these boundary conditions is a crucial step when developing viable interventions. To this end, we examined how gratitude disposition, self-esteem, and optimism relate to the subjective well-being (SWB) and psychological well-being (PWB) of Japanese individuals.

**Methods:**

Multivariate regression analysis was employed to quantify the unique relationships between the three potential intervention targets and both SWB and PWB, while accounting for the influence of other variables. Participants (*N* = 71) also engaged in a 4-week experience sampling study to explore how gratitude, self-esteem and optimism shape the link between momentary affective states in everyday life and evaluations of day satisfaction.

**Results:**

Multivariate regression analysis revealed that self-esteem was predominantly more strongly associated with SWB compared to gratitude disposition, whereas gratitude disposition was more strongly associated with the PWB dimensions, particularly personal growth, positive relations with others and purpose in life. Experience sampling data indicated that while both gratitude disposition and self-esteem moderated the association between momentary positive affect and day satisfaction evaluations, they did so in opposite ways; greater gratitude disposition strengthened the association, while greater self-esteem weakened it.

**Conclusions:**

Overall, the current results suggest that while gratitude, self-esteem, and optimism influence individual well-being as a whole, they likely play distinct roles in facilitating SWB and PWB in the studied cohort.

**Supplementary Information:**

The online version contains supplementary material available at 10.1186/s40359-024-01606-y.

## Background

Devising interventions to effectively promote individual well-being based on sound scientific evidence has been a major goal in the field of positive psychology [1]. Positive psychology interventions have been applied in both clinical and nonclinical studies, in line with the notion that psychology research should not only aspire to help the ill but also look for ways to support healthy individuals to thrive [2]. These interventions typically aim to improve well-being (as assessed by means of psychological scales) by requesting that participants undertake activities that overtly or covertly focus their attention on certain aspects of their daily lives (i.e., the intervention target), such as the emotions they experience and the behaviors they exhibit. Engaging with the designated activity is expected to lead to readjustments in how participants perceive and cognize about that aspect of their lives, which may result in positive changes in outcome variables of interest. Common activities include writing gratitude letters [3, 4] or deliberately engaging in behaviors such as performing acts of kindness [5]. These activities are often scheduled to be performed with a fixed frequency (e.g., once a week) for a certain period of time (e.g., 6 weeks). Effects are assessed at the end of the intervention period and at subsequent follow-ups (e.g., 3 months later).

Initial meta-analyses have indicated that positive psychology interventions can be effective in enhancing individual well-being broadly defined [6, 7]. However, more recent analyses have shown that overall effects are likely to be small to moderate [8, 9]. What has become increasingly evident is that positive psychology interventions do not benefit everyone equally; their effectiveness is subject to the influence of various parameters [10], including participants’ age [7] and the intensity of the intervention [6, 9]. Most importantly, a growing body of evidence has shown that the cultural context surrounding the individuals undergoing the intervention can also act as an important moderator, highlighting the need to personalize interventions to fit specific attributes of the individuals partaking in the activities [11] and develop culturally competent interventions that properly address culture-specific aspects that may affect intervention outcomes in critical ways [12]. For instance, results from a 6-week intervention study where Anglo-American and Asian-American (predominantly foreign-born) individuals engaged in writing gratitude letters or thinking optimistically about the future showed that, in contrast with Anglo-American participants, Asian-American participants in both treatment conditions did not enjoy gains in life satisfaction at the end of the intervention [13]. The authors hypothesized that cultural differences might have accounted for the discrepancies observed in the results: while more individualistic Western cultures emphasize the importance of self-improvement and agency and frame “happiness”, or alternatively a state of greater well-being, as a goal to be pursued and achieved individually, more collectivistic East Asian cultures put greater emphasis on the harmonious interdependence between the self and others [14].

In another study, participants from the USA and South Korea took part in a 6-week intervention involving performing acts of kindness and writing gratitude letters [15]. Intervention effects were assessed using an aggregate measure of well-being based on the Satisfaction with Life Scale (SWLS) [16] and the Modified Differential Emotions Scale [17]. Results indicated that South Korean participants who wrote letters of gratitude enjoyed much lower increases in well-being than did their USA counterparts; in contrast, performing acts of kindness resulted in similar levels of improvement in both groups. The authors reasoned that owing to cultural norms, South Korean individuals may be more likely to experience conflicting emotions in situations associated with gratitude (such as guilt or indebtedness toward the benefactor), which may have restrained the impact the intervention had on the well-being of South Korean participants who primarily engaged with the gratitude activity compared to the kindness activity. The authors further conjectured that the differences observed across groups on self-reported evaluations regarding effort exerted when performing the intervention activities (US participants indicated more effort) might reflect underlying intergroup differences concerning how individuals conceptualize positive activities and the consequences that follow: while Americans cognize that “happiness” is primarily the result of personal effort, South Koreans tend to attribute greater weight to good fortune and fate. This hypothesis aligns with a growing body of evidence that highlights the impact of culture-specific factors on conceptualizations of notions such as happiness and life satisfaction across societies [18–21].

While the precise mechanisms behind the observed differences in these studies are yet to be determined, cultural context appears to be the most salient factor hindering East Asian individuals from benefitting as much as their Western peers from traditional positive psychology interventions. Although there is increasing global interest in positive psychology interventions, most research still originates from Western countries [22]. Consequently, most of these studies have largely relied on data collected from individuals rooted in Western cultures. This issue has afflicted not only research in psychology [23] but also the behavioral sciences in general [24]. Although there has been a marked increase in the number of positive psychology intervention studies involving individuals from non-Western cultures [25], a more detailed understanding of how potential intervention targets relate to well-being outcomes in specific groups within this large, heterogeneous and understudied collective is still greatly warranted. Advancing this evidence base will enable the systematic design of interventions tailored to fit the characteristics of the individuals participating in the activities, leading to more inclusive and impactful practices. At the same time, the development of interventions that can more effectively enhance individual well-being within the frameworks of experimental protocols will be key to advance our understanding of both the psychological and neurobiological mechanisms that underlie the state of well-being, as they will enable the manipulation of individual levels of well-being within the framework of experimental protocols. Deepening our comprehension of how potential intervention targets relate to various well-being measures among Japanese individuals is a crucial step towards obtaining an encompassing view of not only which positive psychology interventions are likely to be most effective, but also in determining their appropriateness for a broader range of individuals.

To address this knowledge gap, we examined how 3 psychological constructs that have been frequently associated with individual well-being [6, 26–29], i.e., gratitude, self-esteem, and optimism, relate to 2 conceptualizations of well-being, i.e., subjective well-being and psychological well-being, among Japanese individuals. Moreover, we also examined how gratitude, self-esteem, and optimism moderated the relationship between everyday life momentary affective states and overall judgments of day satisfaction.

The first conceptualization of well-being – subjective well-being (SWB) – centers around commonly held notions of happiness in the West [30, 31]. It consists of a cognitive component encompassing people’s overall evaluations of their lives and an affective component rooted in the emotions and affective states that people experience in their everyday lives [32]. The SWLS [16] is often employed to measure the cognitive component of SWB, while the affective component is assessed using scales such as the Positive and Negative Affect Schedule [33]. The average life satisfaction of a population is strongly influenced by national wealth, but differences across nations can also be explained by cultural factors, such as the degree of individualistic or collectivistic disposition in a society [26, 34].

The second conceptualization of well-being – psychological well-being (PWB) – is structured around basic competencies hypothesized to empower individuals to function optimally, i.e., autonomy, competence and relatedness [31], or autonomy, environmental mastery, personal growth, positive relations with others, purpose in life and self-acceptance [35]. Scales employed to measure PWB and SWB have often been shown to be positively correlated [36, 37], sparking debate over the need for two distinct conceptualizations of well-being [38, 39]. Nevertheless, studies that have thoroughly examined the relationship between PWB and SWB have found that the two conceptualizations do not overlap entirely [40–43]. The notion of PWB is particularly attractive in the context of positive psychology interventions, as it focuses on competencies that can sustainably foster positive affective states. From this perspective, an individual proficient in core PWB competencies would consequently become more likely to experience happiness more frequently [39]. Consistent with this notion, longitudinal studies with participants in Japan [44] and the USA [45] have shown that while future levels of PWB and SWB are primarily determined by their past levels, past PWB also significantly affects future SWB, while past SWB does not seem to influence future PWB.

### Gratitude

Gratitude disposition has been associated with various well-being indicators [46] and numerous studies have used gratitude interventions to attempt to enhance individual well-being. Gratitude is typically defined as the emotional response that arises when one recognizes and values a benefit that is attributed to external enablers, often the actions of other people, or as a broader sense of appreciation for the positive things in life [47–49]. Gratitude interventions typically involve simple activities such as writing personal letters of appreciation, e.g., [4], or keeping a journal of experiences that evoked gratitude, e.g., [50]. While meta-analyses suggest that gratitude interventions can positively affect metrics of well-being [28, 48, 51], two studies with Japanese participants highlight potential cross-cultural discrepancies in intervention outcomes. In a 4-week intervention study with Japanese individuals working at a local government institution [52], participants were randomly asked at the end of each week to either make a list of up to 5 coworkers to whom one had felt grateful in the past week (gratitude group) or a list of up to 5 personal or work-related events that had taken place in the past week (control group). Curiously, no significant differences were observed across groups in any of the outcome variables of interest; however, while gratitude-related feelings and positive affect grew over time in both groups, such an effect was not observed in the scores for SWLS and the Subjective Happiness Scale (SHS, [53]). In another study, Japanese college students were randomly assigned to either a gratitude group, where they were daily asked to write a list of up to 5 things or people they had felt grateful for, or a control group, where they were only requested to perform daily self-assessments regarding things such as perceived stress [54]. After 2 weeks, while students in the gratitude group showed improved academic motivation [55] compared to the control group, no differences across groups were observed in the SWLS scores. These findings suggest that gratitude interventions might affect well-being differently in Japanese individuals; more broadly, they could be signaling that traditional SWB scales, such as the SWLS and the SHS, might not appropriately capture the impact such interventions have on individuals from such populations.

### Self-esteem

Self-esteem is a psychological construct centered on one’s overall sense of personal worth and confidence in one’s own abilities [56]. Evidence from a meta-analysis indicates that self-esteem can be enhanced by means of behavioral interventions [57]. Despite the intuitive appeal it enjoys with the general public, scholars have debated the extent of the presumed ties between self-esteem and various positive life outcomes [58, 59]. Nevertheless, even critics of the view that self-esteem is an overarching driver of positive life outcomes acknowledge its association with subjective happiness [59, 60]. Largely consistent with that, self-esteem (operationalized as satisfaction with self) was found to be correlated with satisfaction with life in a large sample of respondents from 31 countries [26]. The sample encompassed a wide range of nations with varying scores on an individualism-collectivism scale [61], from more individualistic nations (e.g., USA, Netherlands, Canada) to more collectivistic ones (e.g., Bangladesh, Korea, Japan). This diversity enabled further exploration of whether the strength of the connection between self-esteem and life satisfaction was related to differences in individualism-collectivism scores across nations. Results indicated that in countries with stronger collectivistic orientations (lower individualism-collectivism scores), the link between self-esteem and life satisfaction was less pronounced than in countries with stronger individualistic orientations (higher individualism-collectivism scores), suggesting that cultural values may influence the relationships between psychological constructs and metrics of well-being. Curiously, among the 31 countries in the sample, Japan and South Korea were the only nations where most respondents evaluated both their life satisfaction and self-esteem to be below the neutral point (4) on a scale from ‘terrible’ (1) to ‘delightful’ (7). While these findings should be considered in the context of each nation’s specific circumstances during the years of data collection, they are consistent with the notion that holding positive views about the self is not as valued or common in collectivistic societies compared to more individualistic ones, such as North American societies [62]. Cultural factors can broadly influence how people evaluate self-construals [63] and, consequently, how they relate to metrics of well-being, making it critical to clarify culture-specific relationships when devising positive psychology interventions.

### Optimism

Optimism is the general tendency to expect favorable outcomes in the future, even in the face of adversity [64]. It has been associated with a host of desirable physical [65] and psychological [64] health outcomes, as well as adaptive behaviors [66], particularly in medical contexts, where one’s optimism can regulate coping styles adopted in the presence of stressors [67]. Interventions targeting optimism have largely relied on the Best Possible Self activity [68] or some variant of it, where people are requested to think about their future life (“Imagine that everything has gone as well as it possibly could”) and then write down their reflections. Meta-analyses have indicated that, on the whole, such interventions primarily affect one’s optimism [69], although effect sizes may vary depending on how outcome variables are specifically assessed [70, 71]. Optimism interventions have also been shown to be able to improve metrics of well-being operationalized around core aspects of SWB, i.e., happiness, positive affect, negative affect, and life satisfaction [27, 71]. While the cultural background of participants is often mentioned as a potentially critical moderator of individual differences in optimism [64, 69], thus far, most studies have relied on data collected from individuals rooted in Western societies. Only a few studies have examined cross-cultural differences between the construal of optimism and pessimism with respect to outcome variables of interest, such as life satisfaction, e.g., [72], and only a handful in the context of positive psychology interventions; of all studies included in the meta-analysis by Carrillo et al. (26 studies) and Heekerens and Eid (34 studies), only 4 involved individuals from Asian countries.

### Current study

Our primary goal was to quantify the unique relationships between the three potential intervention targets and both SWB and PWB while accounting for the influence of the other variables using multivariate regression analysis. This dataset was collected before participants took part in the experience sampling phase of the study. Here, SWB was operationalized using the SWLS and the SHS, whereas PWB was operationalized using the 42-item version of the psychological well-being scale ([PWBS, [35]). Given the known association between personality dimensions and both SWB and PWB [73–75], we first examined whether gratitude, self-esteem and optimism could account for variations in well-being scores among Japanese individuals over and above the explanatory power of the Big Five personality dimensions. Next, we sought to explore the relationships between gratitude, self-esteem, and optimism with SWB and PWB. Studies reporting enhancements in SWB among non-Western individuals following participation in positive psychology intervention studies are very limited. In addition, gratitude has been shown to promote prosocial behaviors such as helping [76], impact meaning in life [77, 78] and improve self-acceptance [79]. Based on these results, we hypothesized that among Japanese individuals, gratitude disposition would have a more pronounced association with PWB than SWB. Furthermore, since self-esteem has consistently been linked to SWB indicators, particularly life satisfaction, we anticipated it would predominantly account for variance in SWB. However, we expected this association to be less pronounced than in more individualistic societies due to the more collective nature of our sample [61] and the reduced emphasis Japanese individuals place on positive self-evaluations [62]. Because self-esteem was found to be positively correlated with all 6 dimensions of the PWBS in a Japanese sample (Pearson's r ranging from 0.433 to 0.714, [80]), we hypothesized that it would show an overall association with PWB as well.

Optimism has been shown to be related to life satisfaction among North American [81] and Japanese individuals [82]. Because it has been shown to predict life satisfaction even when accounting for other factors [83], we hypothesized that optimism would be associated with SWB in the current sample. Optimism has been shown to be correlated with meaning in life among Peruvian students [84], a concept akin to the PWB’s purpose in life dimension. Thus, we posited there was going to be a link between optimism and PWB in our sample as well.

In addition, we examined how gratitude, self-esteem, and optimism shape the link between everyday life momentary affective states and day satisfaction evaluations. Although the notion that SWB is structured in terms of affective (i.e., positive and negative affect) and cognitive (i.e., life satisfaction) components is widely accepted, the exact way these primary components combine to form the construct of SWB remains an open question [85]. Models positing life satisfaction as a primary outcome of positive and negative affect have been shown to be empirically viable [86]; consistent with that, emotions experienced in everyday life have been shown to be associated with judgments of satisfaction and meaning [87]. Inspired by these findings, and to complement the results from the cross-sectional data, the same participants took part in a 4-week experience sampling data collection. Based on that data, we empirically examined whether and how gratitude, self-esteem and optimism shape the link between momentary affective states in everyday life and end-of-day judgements of day satisfaction, i.e., the subjective evaluation of how good or bad a day was. Considering the scarcity of prior studies addressing this issue using Western samples or otherwise, we did not make any a priori predictions. However, if the effects observed across the three variables of interest were found to be non-uniform, we hypothesized that this would highlight the significant point that, in the studied cohort, their distinct contributions to individual well-being extend beyond self-reported data gathered in a laboratory setting.

## Methods

### Participants

Seventy-seven participants signed up to the study via an online recruiting system. Recruitment occurred during the months of November 2021 (data collection performed during the months of November/December 2021) and December 2021 (data collection performed during the months of January/February 2022). Participants were requested to attend a lab session (in person or online) where they received orientation about the study and provided answers to various sociodemographic questions and to the items of the psychological scales. Each participant received JPY 1,000 for attending the session. During the following 4-week period when the experience sampling (ESM) data were collected, 196 signals were sent to each participant (JPY 50 was paid for each response). To further improve response compliance, participants were told that an additional bonus of JPY 1,000 would be paid for every week they were able to reply to “most of the signals”; no further details about the weekly bonus were disclosed to prevent excessive influence in response behaviors (participants were not informed about their weekly response performance during the study). The weekly bonus was paid at the end of the study for each week where at least 70% of the signals were responded. Participants could receive a monetary reward of up to JPY 14,800 for partaking in the study.

Four participants (ages 39.6 years to 60.7 years old) who did not belong to the age group of interest (20–35 years old) and 2 participants who declared to be undergoing psychiatric treatment at the time of the study were excluded from the analyses. The final sample consisted of 71 participants (41 identified as male, 30 as female), with a mean age of 23.7 years old (SD = 2.3, range 20.7–34.1). The median and the mode of the ages in this cohort were 23.4 and 22.2 years old, respectively, indicating that the distribution leaned heavily toward the younger end of the age range. Table 1 summarizes the demographic characteristics of the participants.

**Table 1:** Demographic characteristics of the participants. All participants were native Japanese speakers residing in Japan at the time of the study

Participant *N*		71	
Gender			
	Female	30	41%
	Male	41	58%
Age			
	20 to 29	69	97%
	30 to 39	2	3%
Currently a college/graduate student?			
	Yes	64	90%
	No	7	10%
Personal income			
	0–100,000 yen	57	80%
	100,001–200,000 yen	12	17%
	200,001–300,000 yen	2	3%
	300,001–400,000 yen	0	0%
	400,001–500,000 yen	0	0%
	> 500,000 yen	0	0%

### Procedure and materials

Participants received a detailed explanation about the study in the lab session, including instructions regarding the procedures involved in the ESM phase of the study. Participants were reminded that all data collected during the study would be anonymized before analysis. Those who agreed to participate were requested to fill in an informed consent form. Participants then used their cellphones to register to the online system that was used to collect data during the 4-week ESM phase of the study (Exkuma, Japan Experience Sampling Method Association). Participants were briefed about the questions that would be asked during the 4-week ESM phase of the study and instructed on how to record their responses in the system. For 4 weeks, signals were sent to the participants’ cellphones, prompting them to answer the questions. Six signals (affective state signals, 168 in total) were sent between 8:00 am and 10:00 pm at random times, customized for each participant, and 1 additional signal (daily evaluation signals, 28 in total) was sent every night at a fixed time (8:15 pm). The minimal interval between affective state signals was 80 min; participants were told that affective state signals had to be replied within 90 min for the system to record their answers. No constraints were imposed on the daily evaluation signals, but participants were encouraged to try to reply within the same day the signal was sent as much as possible.

Every time participants were prompted by an affective state signal, they were requested to report (i) their current location (home/college campus/part-time job/in transit/other, outside), and (ii) the number of known and (iii) unknown people in a 5-m radius (0/1/2/3/4/5/6 or more) [88]. They were then asked to (iv) evaluate their current mood in terms of 5 positive sentences, namely, “I’m happy”, “I’m relaxed”, “I feel energized”, “I feel merry”, “I feel cheerful”, using 1 = “strongly agree” to 6 = “strongly disagree”, and if they could find a reason for why they were feeling like that, to (v) succinctly describe it (otherwise, declare “nothing in particular” or some equivalent expression). Next, they were asked to (vi) evaluate their mood in terms of 5 negative sentences, namely, “I’m sad”, “I’m angry”, “I feel depressed”, “I feel tired”, “I’m worried”, and if they could find a reason for why they were feeling like that, to (vii) succinctly describe it (otherwise, declare “nothing in particular” or some equivalent expression). When prompted by a daily evaluation signal, participants were requested to (a) choose the option that best described the weather at their current location (rainy/rainy sometimes sunny/cloudy/sunny) and then provide a rating of day satisfaction, similar to [89], by rating the sentences (b) “Overall, it was a good day” and (c) “Overall, it was a bad day” using 1 = “strongly agree” to 6 = “strongly disagree”. Signals began to be sent on the day following the lab session.

After the explanation about the ESM phase of the study, participants were requested to write a list of (1) highly positive events and (2) highly negative events experienced thus far in their lives, followed by a list of (3) highly positive events and (4) highly negative events experienced in the past year [90]. They were given 3 min to complete each of the lists (data not reported). Participants then answered a questionnaire that included, among other things, sociodemographic questions (e.g., monthly income, source of income, whether they were living by themselves, with their parents, etc.), self-evaluations on current happiness (0: least happiest possible, 10: most happiest possible), stress levels (0: no stress at all, 10: extremely stressed), and time spent with family, friends and alone on a typical day during the previous week [91] (see Supplementary Information 1 for the full list of questions). Scale items were also included in the questionnaire (see Measures below for details). A subset of the scales was collected at the end of the 4-week period and every 4 weeks for the following 6 months (data not reported).

## Measures

### Psychological scales

The descriptive statistics regarding the data from the psychological scales are summarized in Table 2.

**Table 2:** Descriptive statistics of the psychological scales. Range refers to the sample range

		*N* = 71		
		*M*	*SD*	Range
NEO-FFI	1 Neuroticism	27.87	9.18	[3–47]
	2 Extraversion	25.18	6.80	[11–40]
	3 Openness	30.82	6.56	[20–46]
	4 Agreeableness	30.52	6.05	[15–47]
	5 Conscientiousness	26.38	7.38	[11–41]
	6 Positivity Scale (P-Scale)	25.63	6.18	[9–40]
	7 Self-Esteem (SE)	26.07	5.63	[13–40]
	8 Satisfaction With Life Scale (SWLS)	20.28	5.78	[5–34]
	9 Revised Life Orientation Test (LOT-R)	15.14	2.74	[8–21]
PWBS	10 Autonomy	28.86	6.38	[14–49]
	11 Environmental Mastery	29.21	6.69	[16–47]
	12 Personal Growth	37.31	5.75	[23–49]
	13 Positive Relationships with Others	34.46	7.28	[13–46]
	14 Purpose in Life	31.46	5.92	[18–47]
	15 Self-Acceptance	30.56	7.53	[13–49]
	16 Gratitude Questionnaire (GQ-6)	31.77	5.61	[18–42]
	17 Subjective Happiness Scale (SHS)	4.69	1.25	[1–7]
	18 CES-D	14.80	9.83	[0–40]

*NEO Five-Factor Inventory-3 (NEO-FFI).* The 60 items of the Japanese translation of the NEO-FFI [92] were used to obtain scores of neuroticism (N), extraversion (E), openness to experience (O), agreeableness (A), and conscientiousness (C). Respondents evaluated each item using a 5-point scale (1 = “strongly disagree” to 5 = “strongly agree”). Cronbach’s alpha was employed to assess internal consistency; in the current sample, the alphas were 0.869 (N), 0.777 (E), 0.722 (O), 0.709 (A), and 0.815 (C).

*Positivity Scale (P-Scale).* Positive orientation [93] was assessed using an 8-item questionnaire designed to measure one’s general tendency to embrace a positive outlook about oneself, one’s life and one’s future [94]. Positive orientation is hypothesized to be a common latent construct underlying self-esteem [56], satisfaction with life [16], and optimism [95]. We employed the Japanese translation of the scale provided by the first author of the original study (personal communication). Respondents evaluated each item using a 5-point scale (1 = “strongly disagree” to 5 = “strongly agree”). Cronbach’s alpha in the current sample was 0.861. The four-week test–retest reliability was assessed using Pearson correlation based on the responses from 67 participants who returned the questionnaire at the end of the ESM phase; in the current sample, r = 0.684.

### Rosenberg’s Self-Esteem (SE)

Self-esteem was assessed using a 10-item questionnaire originally developed to measure the extent to which adolescents are overall contented with themselves [56]. We employed the Japanese translation of the scale developed by Mimura and Griffiths [96]. Respondents evaluated each item using a 4-point scale (1 = “strongly disagree” to 4 = “strongly agree”). Cronbach’s alpha was 0.881, and the four-week test–retest reliability was r = 0.784.

### Satisfaction With Life Scale (SWLS)

Satisfaction with life was measured using a 5-item scale designed to assess subjectively perceived levels of global life satisfaction [16]. We employed the Japanese translation of the SWLS available on the website of the first author of the original study. Respondents evaluated each item using a 7-point scale (1 = “strongly disagree” to 7 = “strongly agree”). Cronbach’s alpha was 0.826, and the four-week test–retest reliability was r = 0.754.

### Revised Life Orientation Test (LOT-R)

Optimism was assessed using the 10 items of the LOT-R [95]. The aggregate score was used in the analysis after reverse scoring the answers to the 3 negatively worded items and adding them to the sum of the answers to the 3 positively worded items, although the negatively and positively worded items can be employed separately to yield scores of pessimism and optimism, respectively. Participants responded to the 4 filler items as well. We employed a Japanese translation of the scale [97]. Respondents evaluated each item using a 4-point scale (1 = “strongly disagree” to 4 = “strongly agree”). Cronbach’s alpha for the 3 positive items, 3 negative items and all 6 items combined were 0.589, 0.312, and 0.603, respectively. Data for the four-week test–retest were not collected, but results from a similar sample have shown that the scale has good reliability (r = 0.82, three-week test–retest, [97]).

### Psychological Well-Being Scale (PWBS)

The 42-item version of the PWBS was used to assess self-reported psychological well-being [35]. The scale employs 7 items to measure each of the 6 dimensions (autonomy, environmental mastery, personal growth, positive relations with others, purpose in life and self-acceptance). We employed the Japanese translation of the scale used in [98]. Items were intermixed, and respondents evaluated each item using a 7-point scale (1 = “strongly disagree” to 7 = “strongly agree”). Cronbach’s alphas were 0.690 (autonomy), 0.796 (environmental mastery), 0.723 (personal growth), 0.833 (positive relations with others), 0.617 (purpose in life), and 0.843 (self-acceptance). The four-week test–retest reliability r values were 0.793 (autonomy), 0.755 (environmental mastery), 0.785 (personal growth), 0.874 (positive relations with others), 0.814 (purpose in life), and 0.796 (self-acceptance).

### Gratitude Questionnaire (GQ-6)

Dispositional gratitude was measured using the 6-item questionnaire developed by McCullough et al. [99]. We employed the Japanese translation of the scale developed by Kobayashi [100]. Respondents evaluated each item using a 7-point scale (1 = “strongly disagree” to 7 = “strongly agree”). Cronbach’s alpha was 0.784. Data for the four-week test–retest were not collected, but results from a similar sample have shown that the scale has good reliability (r = 0.86, four-week test–retest, [101]).

### Subjective Happiness Scale (SHS)

This 4-item questionnaire was developed to directly assess individual levels of global happiness [53]. Happiness is thought to be closely related to the affective component of subjective well-being, i.e., the predominance of positive/pleasant affect over negative/unpleasant affect [30]. Respondents evaluated each item using a 7-point scale; anchor labels varied depending on the item: 1 = “not a very happy person” to 7 = “a very happy person”, 1 = “less happy” to 7 = “more happy”, 1 = “not at all” to 7 = “a great deal”. We employed the Japanese translation of the scale developed by Shimai et al. [102]. Cronbach’s alpha was 0.884. Data for the four-week test–retest were not collected, but results from a similar sample have shown that the scale has good reliability (r = 0.86, five-week test–retest, [102]).

### Center for Epidemiologic Studies Depression Scale (CES-D)

This is a 20-item questionnaire developed to assess symptoms of depression in the general population [103]. Respondents evaluated each item according to the duration of symptoms in the previous week using 0 = “rarely or none”, 1 = “1 to 2 days”, 2 = “3 to 4 days”, and 3 = “5 or more days”. We employed the Japanese translation of the scale [104]. Cronbach’s alpha in the current sample was 0.894, and the four-week test–retest reliability was r = 0.771.

### Experience sampling method

Responses for the 5 momentary positive affect items and the 5 momentary negative affect items resulting from a single signal were averaged to form a composite positive and negative affect score, respectively. The average Cronbach’s alpha across participants for the positive and negative ratings were 0.875 and 0.636, respectively, indicating that the within-person internal consistency of momentary positive affect responses was on average higher than the internal consistency of momentary negative affect responses. These composite scores were grouped by day and further averaged, resulting in one mean momentary positive affect score (PA) and one mean momentary negative affect score (NA) for each of the days of the ESM phase for each participant. The scores were then paired with the “good day” (GD) and “bad day” (BD) responses to the daily evaluation signals. Both PA and NA and GD and BD were significantly negatively correlated (Spearman’s rank correlation rho = -0.591 and rho = -0.831, respectively). Days in which the mean affect scores could not be computed, due to the complete lack of responses to the affective state signals sent on that day or the absence of a daily evaluation signal response, were eliminated from the dataset.

### Statistical analyses

IBM SPSS Statistics (version 28) and MATLAB (R2021b Update 3) were used to preprocess and analyze the cross-sectional data. Personality has been associated with a range of life outcomes closely associated with the notion of well-being [105, 106]; therefore, we first examined whether gratitude, optimism and self-esteem could add explanatory power to measures of well-being beyond what is already accounted for by personality dimensions in the current sample. Failure to do so would suggest that the respective variable had little potential to serve as a target in a well-being intervention. Hierarchical regressions were performed with SWLS, SHS and the 6 dimensions of PWB as dependent variables. The five personality dimensions were entered as predictor variables at step 1 of the hierarchical regression; at step 2, one of the variables of interest, i.e., gratitude (GQ-6), self-esteem (SE), or optimism (LOT-R), was further added to the model as a predictor.

Multivariate regression analysis was employed to simultaneously examine the relationships between gratitude, self-esteem, and optimism with metrics of SWB and PWB. We also included CES-D scores as an outcome variable to explore relationships with depressive symptom intensity. Specifically, we performed a multivariate regression analysis with CES-D, SWB and the 6 PWBS dimensions as outcome variables and gratitude (GQ-6), self-esteem (SE), optimism (LOT-R), current stress, and gender (operationalized as a dummy variable with female = 0, male = 1) as predictor variables while additionally controlling for age and income (0–100,000 yen = 1, 100,001–200,000 yen = 2, …, > 500,000 yen = 6). These additional variables were included based on evidence indicating their influence on individual well-being differences [107–112].

Hierarchical linear models implemented using HAD version 17.2 [113] were employed to analyze the data collected in the experience sampling phase. Signal responses (level-1) were nested within participants (level-2) to analyze the associations between PA (level-1 predictor variable) and judgments of “Overall, it was a good day” (level-1 outcome variable), and associations between NA (level-1 predictor variable) and judgments of “Overall, it was a bad day” (level-1 outcome variable). To disaggregate level-1 and level-2 effects [114], PA and NA were person-mean-centered (i.e., the mean PA/NA of a participant was subtracted from all the respective daily scores). Furthermore, to examine how those associations were affected by gratitude, self-esteem and optimism, each of those individual scores was added as a level-2 predictor variable, together with a factor to account for an interaction between them and the PA (NA) scores. Level-2 predictors were grand-mean centered to facilitate interpretation of possible interaction results. A random intercept and a random slope for the PA (NA) scores were included in the model to take individual differences in within-participant associations between momentary affect and day evaluations into account and examine potential between-participant differences. For all analyses, statistical significance was set at a threshold of *p* < 0.05. Bonferroni correction for multiple comparisons was employed when assessing the pairwise linear correlation coefficients between the various scales.

## Results

### Psychological scales

Table 3 shows the pairwise linear correlation coefficients (Pearson) between the scores obtained from the self-reported data collected in the lab session. Among the personality dimensions, neuroticism and extraversion have been frequently associated with well-being indicators, particularly those associated with the concept of SWB [115, 116]. Results involving neuroticism and extraversion largely replicated those reported in previous studies. Because most of the scales had a positive flavor, neuroticism was negatively correlated with virtually all of them, in particular self-esteem, SWLS, optimism (LOT-R), environmental mastery, self-acceptance and SHS. Neuroticism has been associated with several mood and physical disorders [117], especially depression [118–120]; in line with previous results, neuroticism was positively correlated with CES-D scores in the current sample. Extraversion, on the other hand, was negatively correlated with CES-D scores and positively correlated with positive orientation, self-esteem, optimism and scales associated with SWB, i.e., SWLS [121, 122] and SHS [123], as well as with all the PWB dimensions [124], except for autonomy and purpose in life. Regarding the other personality dimensions, openness was correlated with SWLS and personal growth, although in both cases, statistical significance did not pass the Bonferroni correction for multiple comparisons. Agreeableness was positively correlated with personal growth, positive relations with others and GQ-6. Conscientiousness was positively correlated with purpose in life.

**Table 3:**
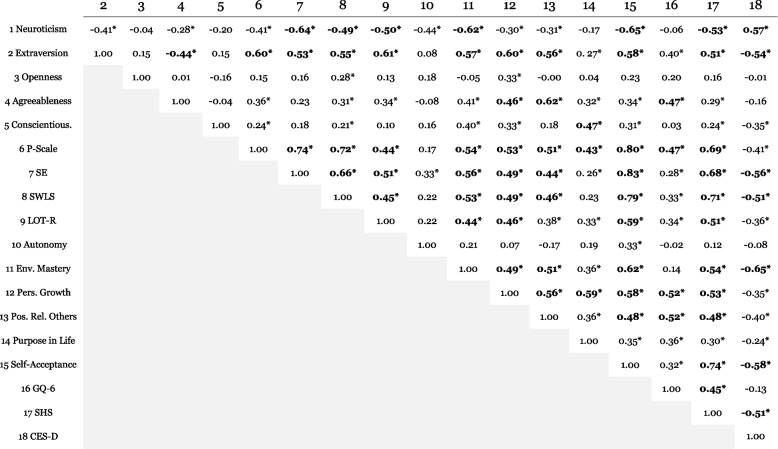
Pairwise linear correlation coefficients (Pearson) between the scales (*N* = 71). Asterisks indicate coefficients that were statistically significant (*p* < .05); values highlighted in boldface were still significant after applying Bonferroni correction for 153 comparisons. Spearman rank correlations were computed when appropriate, i.e., when one or both variables failed to pass the Anderson‒Darling test for normality (*p* < .05)

Positive orientation [93] is based on the constructs of self-esteem, life satisfaction (SWLS) and optimism; therefore, as expected, it showed robust correlation with all three scales. Furthermore, positive orientation was positively correlated with all PWB dimensions (apart from autonomy), GQ-6 and SHS. Aside from the correlations mentioned previously, self-esteem was positively correlated with SWLS, optimism, environmental mastery, personal growth, positive relations with others, self-acceptance and SHS; moreover, it was negatively correlated with CES-D. SWLS was positively correlated with optimism and all PWB dimensions (except for autonomy and purpose in life) as well as SHS, in addition to the correlations mentioned before. Optimism was positively correlated with environmental mastery, personal growth, self-acceptance and SHS, in addition to the correlations mentioned previously. Finally, GQ-6 was positively correlated with SHS, in addition to the links mentioned before.

Results from the hierarchical regressions are summarized in Table 4. Adding gratitude to the regression model initially fed with the personality dimensions significantly explained additional variance of SHS and the PWB dimensions of positive relations with others and purpose in life. Adding self-esteem significantly explained additional variance in the SWLS, SHS, and the PWB dimensions of personal growth, positive relations with others and self-acceptance. Adding optimism significantly explained additional variance of SHS and the PWB dimension of self-acceptance. Overall, the results showed that all three variables could explain variance beyond personality dimensions in at least one metric of SWB and one dimension of PWB. Curiously, in this sample, only self-esteem, but not gratitude or optimism, was found to influence SWLS beyond personality dimensions.

**Table 4:** Summary of hierarchical multiple regressions (*N* = 71) individually testing the incremental validity of Gratitude (GQ-6), Self-Esteem (SE) and Optimism (LOT-R) in predicting PWBS, SWLS and SHS scores (Step 2), controlling for NEO-FFI personality factors (Step 1). The statistical significance of each ∆R^2^ is indicated by *p*

			Step 1		Step 2		Change statistics		
			R^2^	F(5, 65)	R^2^	F(6, 64)	∆R^2^	F(1, 64)	*p*
GQ-6	PWBS	Autonomy	.300	5.577^***^	.301	4.603^***^	.001	.113	.738
		Env. Mastery	.604	19.831^***^	.607	19.494^***^	.003	.528	.470
		Pers. Growth	.602	19.670^***^	.624	17.697^***^	.022	3.717	.058
		Pos. Rel. Others	.575	17.557^***^	.617	17.182^***^	.042	7.087^*^	.010
		Purpose in Life	.340	6.707^***^	.392	6.885^***^	.052	5.468^*^	.023
		Self-Acceptance	.593	18.972^***^	.612	16.482^***^	.019	3.111	.083
	SWLS		.455	10.868^***^	.472	9.546^***^	.017	2.055	.157
	SHS		.408	8.972^***^	.487	10.130^***^	.079	9.826^**^	.003
SE	PWBS	Autonomy	.300	5.577^***^	.305	4.679^***^	.005	.434	.513
		Env. Mastery	.604	19.831^***^	.613	16.870^***^	.009	1.420	.238
		Pers. Growth	.602	19.670^***^	.640	18.930^***^	.038	6.661^*^	.012
		Pos. Rel. Others	.575	17.557^***^	.612	16.794^***^	.037	6.097^*^	.016
		Purpose in Life	.340	6.707^***^	.356	5.901^***^	.016	1.574	.214
		Self-Acceptance	.593	18.972^***^	.778	37.337^***^	.184	53.110^***^	< .001
	SWLS		.455	10.868^***^	.553	13.205^***^	.098	14.011^***^	< .001
	SHS		.408	8.972^***^	.577	14.554^***^	.169	25.534^***^	< .001
LOT-R	PWBS	Autonomy	.300	5.577^***^	.308	4.739^***^	.007	.683	.412
		Env. Mastery	.604	19.831^***^	.604	16.277^***^	.000	.012	.913
		Pers. Growth	.602	19.670^***^	.606	16.392^***^	.004	.601	.441
		Pos. Rel. Others	.575	17.557^***^	.575	14.446^***^	.001	.104	.749
		Purpose in Life	.340	6.707^***^	.356	5.899^***^	.016	1.564	.216
		Self-Acceptance	.593	18.972^***^	.626	17.853^***^	.033	5.577^*^	.021
	SWLS		.455	10.868^***^	.461	9.141^***^	.006	.731	.396
	SHS		.408	8.972^***^	.457	8.962^***^	.048	5.682^*^	.020

^*^ *p* < .05, ** *p* < .01, *** *p* < .001

Having established that gratitude, self-esteem, and optimism can influence SWB and PWB at a very basic level, we sought to quantitatively assess the extent of their influences on the metrics of well-being, along with current level of stress and gender, controlling for age and income. The results from the multivariate regression analysis are summarized in Table 5. Unstandardized regression coefficients (B) reveal the sign of the relationship, whether positive or negative; effect sizes were computed in terms of partial eta squared values (${\upeta }_{{\text{p}}}^{2}$) to gauge the strength of the relationship.

**Table 5:** Multivariate regression results (*N* = 71). Outcome variables are displayed in the columns, and predictor variables are displayed in the rows. Statistically significant results (*p* < .05) are highlighted in boldface—unstandardized coefficients (B), and respective effect sizes in terms of partial eta squared (${\upeta }_{{\text{p}}}^{2}$); 95% confidence intervals are in square brackets. Goodness-of-fit is indicated by the adjusted R^2^

		CES-D	PWBS						SWLS	SHS
			Autonomy	Env. Mastery	Pers. Growth	Pos. Rel. Others	Purpose in Life	Self-Accept		
	adjR^2^	.532	.104	.398	.432	.432	.145	.791	.571	.599
Gender (Fem. = 0, Male = 1)	${\eta }_{p}^{2}$	.001	.000	.001	.020	.001	.000	.038	**.066**	.000
	B	-.503 [-3. 943, 2.938]	-.026 [-3.115, 3. 063]	-.245 [-2.899, 2.408]	-1.263 [-3.477, .950]	.339 [-2.468, 3.145]	-.222 [-3.020, 2.575]	-1.395 [-3.154, .363]	**-2.049** [-3.985, -.113]	-.016 [-.422, .389]
Current stress	${\eta }_{p}^{2}$	**.300**	.011	**.158**	.003	**.102**	.013	**.171**	**.122**	.018
	B	**1.905** [1.173, 2.637]	.278 [-.379, .935]	**-.972** [-1.536, -.407]	-.110 [-.581, .361]	**-.799** [-1.396, -.202]	.266 [-.329, .861]	**-.675** [-1.049, -.301]	**-.609** [-1.021, -.197]	-.046 [-.132, .040]
GQ-6	${\eta }_{p}^{2}$	.000	.011	.000	**.201**	**.283**	**.122**	.020	.031	**.103**
	B	-.025 [-.341, .292]	-.119 [-.404, .165]	.010 [-.234, .254]	**.405** [.201, .609]	**.644** [.385, .902]	**.382** [.124, .639]	.092 [-.070, .254]	.126 [-.052, .304]	**.050** [.013, .087]
SE	${\eta }_{p}^{2}$	**.081**	**.067**	**.074**	**.081**	.023	.014	**.533**	**.249**	**.289**
	B	**-.447** [-.826, -.068]	**.363** [.022, .703]	**.328** [.036, .621]	**.288** [.044, .532]	.190 [-.120, .499]	.148 [-.160, .457]	**.822** [.628, 1.016]	**.488** [.274, .701]	**.113** [.068, .158]
LOT-R	${\eta }_{p}^{2}$	**.060**	.005	.049	.015	.003	.007	**.075**	.008	.053
	B	**-.744** [-1.482, -.005]	.196 [-.467, .859]	.513 [-.057, 1.082]	.236 [-.239, .711]	.131 [-.471, .734]	.204 [-.396, .804]	**.427** [.049, .804]	.151 [-.264, .566]	.082 [-.005, .169]

Results showed that among the PWB dimensions, gratitude was a predictor of personal growth (B = 0.405, ${\upeta }_{{\text{p}}}^{2}$ =0.201), positive relations with others (B = 0.644, ${\upeta }_{{\text{p}}}^{2}$ =0.283), and purpose in life (B = 0.382, ${\upeta }_{{\text{p}}}^{2}$ =0.122). Gratitude was also a predictor of SHS (B = 0.050, ${\upeta }_{{\text{p}}}^{2}$ =0.103). In contrast, self-esteem was a predictor of CES-D (B = -0.447, ${\upeta }_{{\text{p}}}^{2}$ =0.081) and the PWB dimensions of autonomy (B = 0.363, ${\upeta }_{{\text{p}}}^{2}$ =0.067), environmental mastery (B = 0.328, ${\upeta }_{{\text{p}}}^{2}$ =0.074), personal growth (B = 0.288, ${\upeta }_{{\text{p}}}^{2}$ =0.081) and self-acceptance (B = 0.822, ${\upeta }_{{\text{p}}}^{2}$ =0.533). In addition, self-esteem was also found to be a predictor of SWLS (B = 0.488, ${\upeta }_{{\text{p}}}^{2}$ =0.249) and SHS (B = 0.113, ${\upeta }_{{\text{p}}}^{2}$ =0.289). Optimism was a predictor of CES-D (B = -0.744, ${\upeta }_{{\text{p}}}^{2}$ =0.060) and PWB self-acceptance (B = 0.427, ${\upeta }_{{\text{p}}}^{2}$ =0.075), but no association was found with metrics of SWB. Current level of stress was a predictor of CES-D (B = 1.905, ${\upeta }_{{\text{p}}}^{2}$ =0.300), PWB environmental mastery (B = -0.972, ${\upeta }_{{\text{p}}}^{2}$ =0.158), positive relation with others (B = -0.799, ${\upeta }_{{\text{p}}}^{2}$ =0.102), self-acceptance (B = -0.675, ${\upeta }_{{\text{p}}}^{2}$ =0.171) and SWLS (B = -0.609, ${\upeta }_{{\text{p}}}^{2}$ =0.122). We also observed an effect of gender on SWLS (B = -2.0.49, ${\upeta }_{{\text{p}}}^{2}$ =0.066); because female individuals were coded as 0 and male individuals were coded as 1, this result indicates that gender had a negative impact on the SWLS of male individuals in this sample.

### ESM data

Six participants failed to reply to more than 50% of the affective state signals, so their data were excluded from the analysis (3 male and 3 female participants). They responded on average to only 26.2% of the affective state signals (SD = 0.129) and 49.4% of the daily evaluation signals (SD = 0.369). Compliance among the remaining 65 participants was visibly much better; they responded on average to 85.5% of the affective state signals (SD = 0.086) and 94.2% of the daily evaluation signals (SD = 0.070), resulting in a dataset of 9,340 affective state signals and 1,714 daily evaluation signals in total. On average, participants were surrounded by 0.976 known people (SD = 0.794, range: 0 to 3.481) and 0.937 unknown people (SD = 0.763, range: 0 to 5.413) when responding to the affective state signals. Across participants, the distribution regarding the place from which the signals were responded was home 59.9% (SD = 0.211), college campus 16.0% (SD = 0.162), part-time job 3.9% (SD = 0.051), in transit 9.9% (SD = 0.072), and other, outside 10.2% (SD = 0.085). Furthermore, on average, participants described a reason for their positive affective state (other than “nothing in particular”) in 65.9% of the affective state signals (SD = 0.347, range 0 to 1) and a reason for their negative affective state (other than “nothing in particular”) in 56.4% of the signals (SD = 0.364, range 0 to 1). Both values were highly correlated across participants (Pearson r = 0.922), indicating that those who declared a reason for their current affective state did that consistently for both positive and negative states.

Because the internal consistency of the momentary negative affect ratings was low compared to the momentary positive affect ratings and the GD and BD were strongly correlated, we focused the analysis on the association between PA and GD only. Multilevel modeling results showed that both within (PA (pm)) and between-participants (PA (gm)), greater mean momentary positive affect was associated with higher GD scores, i.e., days on which greater momentary positive affect was experienced were associated with higher “good day” evaluations (relative to other days), and participants who enjoyed greater momentary positive affect also gave higher “good day” evaluations (relative to other participants). Similarly, participants with greater self-esteem scores (SE) were also associated with higher GD scores. Interaction factors indicated a distinct moderator effect of SE and gratitude (GQ-6): while increasing GQ-6 strengthened the association between PA and GD (positive B), increasing SE had the opposite effect (negative B). Moreover, PA significantly differed across participants, as did the association between PA and GD (see random effects results). Results are summarized in Table 6.

**Table 6:** Associations between mean positive affect scores (PA), gratitude (GQ-6), self-esteem (SE), and optimism (LOT-R) and “good day” evaluations (GD), estimated using hierarchical linear modeling based on the experience sampling data collected over the course of 4 weeks (N = 65). Statistically significant results (*p* < .05) are highlighted in boldface. pm: person-mean centered variables; gm: grand-mean centered variables

		B (SE)	95% CI	t	*p*
Fixed effects					
	PA (pm)	0.899^***^ (0.044)	[0.811, 0.986]	20.561	< .001
	PA (gm)	0.856^***^ (0.083)	[0.690, 1.023]	10.291	< .001
	GQ-6 (gm)	-0.003 (0.010)	[-0.023, 0.017]	-0.305	.761
	SE (gm)	0.026^*^ (0.011)	[0.004, 0.048]	2.323	.024
	LOT-R (gm)	-0.034 (0.018)	[-0.070, 0.003]	-1.854	.069
	PA * GQ-6	0.037^***^ (0.010)	[0.017, 0.056]	3.833	< .001
	PA * SE	-0.016^*^ (0.007)	[-0.030, -0.002]	-2.319	.024
	PA * LOT-R	0.004 (0.016)	[-0.028, 0.035]	0.236	.814
		σ^2^	df	χ^2^	*p*
Random effects					
	Intercept	0.153^***^	60	454.370	< .001
	PA	0.023^*^	61	85.069	.023

*B* Unstandardized regression coefficient, *SE* Standard error, *CI* Confidence interval, *t* t-statistic, *p* p value, σ^2^ variance component, *df* degrees of freedom, χ^2^ chi-square statistic

^*^ *p* < .05, *** *p* < .001

## Discussion

To optimize the effectiveness of positive psychology interventions, it is crucial to take into consideration the underlying structure of well-being in the target population. To address this question, we examined how gratitude, self-esteem and optimism are related to two conceptualizations of well-being among Japanese individuals. As hypothesized, multivariate regression results indicated that gratitude disposition was more strongly associated with the PWB dimensions than the core SWB scales (i.e., SWLS and SHS). Conversely, self-esteem was more strongly associated with SWLS and SHS than PWB, except for the dimension of self-acceptance. These findings suggest an interplay between gratitude and self-esteem that has not been explored in prior studies. While previous research has highlighted the general importance of cultural context in determining outcomes of positive psychology interventions, the current findings offer a deeper insight of how specific psychological constructs relate to well-being measures among Japanese individuals.

Importantly, these results suggest that gratitude and self-esteem play complementary roles as enablers of well-being in the examined sample and underscore the importance of understanding the underlying structure connecting intervention targets to outcomes when designing interventions that cater to the characteristics of a particular cohort. It is illustrative that the GQ-6 did not significantly explain SWLS variance beyond personality factors in the dataset composed mostly by Japanese college students (Table 4), in great contrast with the results of a previous study involving UK college students [125]. This finding dovetails with previous studies involving Japanese participants that failed to detect enhancements in life satisfaction following a gratitude intervention (e.g., [52, 54]). This raises the possibility that among Japanese individuals, and possibly other East Asian populations, gratitude disposition is not strongly linked with metrics of life satisfaction as previously assumed, making gratitude interventions possibly less effective in improving SWB. Indeed, additional analyses based on an expanded dataset collected via a web survey (*N* = 1,024, mean age 45.8 years old (SD = 13.8), range = 20–69, Supplementary Information 2, Table C) indicated that even though GQ-6 is linked with SWLS, its effect is much smaller than that of self-esteem. At face value, these results suggest that self-esteem would be a more viable intervention target if the primary goal is to strengthen feelings of life satisfaction (SWLS) or happiness (SHS). On the other hand, given the stronger associations observed between gratitude and the PWB dimensions, the current results would predict that gratitude interventions should result in improvements related to facets associated with PWB, for instance, positive relations with others.

A dissociation between gratitude and self-esteem was also observed in the interaction results from the hierarchical linear modeling based on the experience sampling data, collected daily over the course of 4 weeks (Table 6). While greater levels of gratitude disposition strengthened the association between momentary positive affect ratings collected during the day and end-of-day judgments of day satisfaction, greater levels of self-esteem had the opposite effect. Interestingly, self-esteem, but not gratitude, had a direct effect on day satisfaction evaluations, suggesting that people high in self-esteem typically assign higher scores of “good day”. Taken both results together, one possible interpretation would be that high self-esteem individuals are less reliant on momentary affective experiences when making judgments of day satisfaction. More broadly, they signalize that gratitude disposition and self-esteem distinctly facilitate individual well-being in the current sample.

What these results suggest in their entirety is that only by understanding the relationships between intervention targets and measures of well-being can positive psychology interventions be optimized to improve the well-being of specific cohorts. It is symptomatic that recent meta-analyses have shown that the effects of positive psychology interventions are not as large as once thought to be [8, 9]. This is largely consistent with the view that person-activity fit [10, 11] across studies is overall low, resulting in suboptimal outcomes. Gaining a better understanding of the impact of cultural factors on intervention outcomes, along other factors such as age, gender and personality, will be crucial to move the field from a one-size-fits all approach toward the practice personalized positive psychology interventions that are based on robust scientific evidence. Tailoring interventions to enhance individual well-being more effectively will also enable the study of the psychological and biological mechanisms underlying the state of well-being in much greater detail.

There are several limitations that must be kept in mind. First and foremost, the links clarified by the current results do not necessarily imply causality. Causal relationships are more reliably established through intervention or longitudinal studies. Evidently, a prerequisite for designing effective interventions is a nuanced understanding of how things are structured in the target population. Furthermore, the results from the multivariate regression analysis, including the effect sizes, are largely dependent on our selection of predictor variables, i.e., gratitude, self-esteem, and optimism. That choice was guided by previous studies and our particular interest in intervention paradigms that are self-applied, scalable, and easy to implement and monitor using digital technologies. Nevertheless, there is no shortage of alternatives [9]; it remains to be verified how gratitude, self-esteem and/or optimism interventions fare against other interventions as enablers of SWB and PWB in this particular population.

Another limitation is that results were largely based on answers retrieved using psychological scales, sometimes based on ratings systems that might not have looked entirely intuitive or natural to the respondents. Such type of data may be especially affected by demand characteristics and other sociodemographic and cultural factors. There is no question that they are a valuable source of information [126] and can provide unique insights into the affective-cognitive processes underlying various psychological constructs [127]; however, future work should seek to corroborate such findings with converging evidence based on data collected using alternative approaches, such as behavioral assessments, physiological recordings or observer-based methods. Experience sampling maximizes ecological validity and provides a practical alternative to minimize dependency on self-reported data collected using psychological scales in laboratory settings.

As the current study showed, experience sampling can provide invaluable insights that cannot be attained by laboratory-based methodologies alone. However, it is important to bear in mind that the results are predicated on the assumption that affective states experienced during the day are associated with end-of-day judgments of day satisfaction. This implicitly assumes a model of SWB in which the causality flows from the affective components of SWB to its cognitive component (i.e., life satisfaction). Although that model has been shown to be a viable model for understanding the internal structure of SWB [86], there are other competing models that also merit consideration [85, 86]. SWB is likely to be a dynamically changing complex construct, therefore, more work is necessary to refine and qualify the current findings, to further clarify under which conditions the current results hold.

One notable limitation of our study is that it overlapped with the COVID-19 pandemic. The unprecedented circumstances and global challenges posed by the pandemic may have influenced participants' responses in ways not typically observed under pre-or post-pandemic conditions. The unique stressors and disruptions during this period may have influenced the results in ways that can only be clarified in the future via replication studies.

Pertinent to the current work, there is the critical question of whether the conceptualizations of SWB and PWB in the psychology literature in general, and as operationalized in the current study in particular, are equally relevant across individuals of different cultural backgrounds [19], or individuals that share the same cultural background but have different sociodemographic characteristics. This is not a trivial matter; without a proper understanding of such nuances, interventions may be inadvertently attempting to optimize the wrong outcome. For instance, it has been shown that owing to culture-specific attitudes and thinking styles, the very act of “seeking happiness” may paradoxically lead to impairments in happiness and well-being; highly motivated happiness seekers were associated with lower well-being in the US (more individualistic society) but with higher well-being in East Asia (more collectivistic societies) [128]. Culturally sensitive scales may allow to more appropriately assess constructs that are integral to the notion of well-being, such as happiness [129]. These more refined measures allow the examination of differences and similarities surrounding the structure of well-being within and across cultures. For instance, recent results employing such measures have raised questions about the appropriateness of life satisfaction as a metric of well-being in more collectivistic societies [130]. Such insights provide a foundational framework upon which positive psychology interventions tailored to specific cultural contexts can be developed.

## Conclusions

The need to effectively promote individual well-being through simple interventions has grown, especially considering the global decline in happiness over the past decade around the world, a trend that persisted throughout the COVID-19 pandemic [131]. Our findings suggest that while gratitude, self-esteem, and optimism influence individual well-being as a whole, they likely play distinct roles as facilitators of SWB and PWB among Japanese individuals. Future studies should capitalize on findings like these to develop more culturally competent positive psychology interventions.

### Supplementary Information

**Supplementary Material 1.**

## Data Availability

The datasets generated during the current study are available from the corresponding author on reasonable request.
