# Supplementary material for: Distinct associations between gratitude, self-esteem, and optimism with subjective and psychological well-being among Japanese individuals
Source: BMC Psychol. 2024 Mar 7;12:130. doi: 10.1186/s40359-024-01606-y (PMC10918921; doi:10.1186/s40359-024-01606-y)
Supplement: Supplementary file 1 — Supplementary Material 1. [file 40359_2024_1606_MOESM1_ESM.pdf]

Supplementary Information

Article title: Distinct associations between gratitude, self-esteem, and optimism with subjective and psychological well-being among Japanese individuals.

Norberto Eiji Nawa[1, 2] & Noriko Yamagishi[1, 3]

[1] Center for Information and Neural Networks (CiNet), Advanced ICT Research Institute, National Institute of Information and Communications Technology (NICT)

[2] Graduate School of Frontier Biosciences, Osaka University

[3] College of Global Liberal Arts, Ritsumeikan University

Corresponding author: eiji.nawa@nict.go.jp

Supplementary Information 1

List of additional questions asked alongside the psychological scale items:

1. Imagine a staircase. The lowest step corresponds to the worst possible life (0) and the highest step corresponds to the best possible life (10). Currently, where do you locate yourself in terms of happiness? (1 to 10)
2. Imagine yourself 5 years from now. Using the same staircase, where do you see yourself in terms of happiness in the future? (1 to 10)
3. Recall yourself 5 years ago. Using the same staircase, where were you in terms of happiness back then? (1 to 10)
4. When you woke up this morning, did you feel well rested? (Yes: 1; No: 2)
5. Use the scale below to evaluate the amount of overall stress you are currently facing in your life. (Not at all: 0; Extremely stressful: 10)
6. Did you feel a lot of stress yesterday? (Yes: 1; No: 2)
7. What is your source of income? (Full-time job, Part-time job, Scholarship, Allowance from parents/family, Savings, Living with parents (little expenses), Other)
8. What is your monthly income? (1: 0-100,000 yen; 2: 100,001-200,000 yen; 3: 200,001-300,000; 4: 300,001-400,000; 5: 400,001-500,000; 6: More than 500,000)
9. What would be your ideal monthly income? (1: 0-100,000 yen; 2: 100,001-200,000 yen; 3: 200,001-300,000; 4: 300,001-400,000; 5: 400,001-500,000; 6: More than 500,000)
10. Do you live... (1: By yourself; 2: With your parents or other family; 3: With people other than parents or other family; 4: In a shared house or dormitory; 5: Other)
11. Indicate the type of dinner you had in the past 7 days:
    a. Ate outside (0 to 7)
    b. Bought a meal from a convenience store (0 to 7)
    c. Cooked myself (0 to 7)
    d. Someone else (e.g., family member) cooked for me (0 to 7)
12. For students:
    a. You are currently in your ... (1: Undergrad 1st year; 2: Undergrad 2nd year; 3: Undergrad 3rd year; 4: Undergrad 4th year; 5: Undergrad 5th year and above; 6: Grad school Master’s course; 7: Grad school PhD course; 8: Other)

  b. In the past 6 months, the courses, and seminars you attended were mostly … (1: In person; 2: Online; 3: A mixture of both; 4: Did not attend courses or seminars, 5: Other)
  c. Evaluate your current motivation towards academic activities (No motivation at all: 1; Highly motivated: 10)

13. For non-students:
  a. What is your educational background? (1: Junior high-school; 2: High-school; 3: Vocational school; 4: Junior college; 5: College/University; 6: Graduate school; 7: Other)
14. In the past week, how many hours did you spend with your family on a typical day? (1: 0 hours; 2: 0-1 hour; 3: 1-2 hours; 4: 2-3 hours; 5: 3-4 hours; 6: 4-5 hours; 7: 5-6 hours; 8: 6-7 hours; 9: 7-8 hours; 10: More than 8 hours)
15. Ideally, how many hours would you like to spend with you family on a given day? (1: 0 hours; 2: 0-1 hour; 3: 1-2 hours; 4: 2-3 hours; 5: 3-4 hours; 6: 4-5 hours; 7: 5-6 hours; 8: 6-7 hours; 9: 7-8 hours; 10: More than 8 hours)
16. In the past week, how many hours did you spend with your friends on a typical day? (1: 0 hours; 2: 0-1 hour; 3: 1-2 hours; 4: 2-3 hours; 5: 3-4 hours; 6: 4-5 hours; 7: 5-6 hours; 8: 6-7 hours; 9: 7-8 hours; 10: More than 8 hours)
17. Ideally, how many hours would you like to spend with your friends on a given day? (1: 0 hours; 2: 0-1 hour; 3: 1-2 hours; 4: 2-3 hours; 5: 3-4 hours; 6: 4-5 hours; 7: 5-6 hours; 8: 6-7 hours; 9: 7-8 hours; 10: More than 8 hours)
18. In the past week, how many hours did you spend alone on a typical day? (1: 0 hours; 2: 0-1 hour; 3: 1-2 hours; 4: 2-3 hours; 5: 3-4 hours; 6: 4-5 hours; 7: 5-6 hours; 8: 6-7 hours; 9: 7-8 hours; 10: More than 8 hours)
19. Ideally, how many hours would you like to spend alone on a given day? (1: 0 hours; 2: 0-1 hour; 3: 1-2 hours; 4: 2-3 hours; 5: 3-4 hours; 6: 4-5 hours; 7: 5-6 hours; 8: 6-7 hours; 9: 7-8 hours; 10: More than 8 hours)
20. Have you engaged in volunteer activities in the past year? (Yes: 1; No: 2)
21. Have you donated money to charity in the past year? (Yes: 1; No: 2)
22. Do you have someone you can count on if you face a big problem? (Yes: 1; No: 2)
23. Do you feel acknowledge by people around you? (Not at all: 1; Very much: 10)

24. Do you smoke? (1: Everyday; 2: Occasionally; 3: No)
25. Do you drink? (1: Everyday; 2: Occasionally; 3: No)
26. Do you exercise regularly to keep in shape? (1: Not at all; 2: When I have time; 3: Regularly once or twice a week; 4: Regularly 3 or more times a week)
27. In the past week, did you experience some type of physical pain (headache, stomachache, etc.) that forced you to take a break? (Yes: 1; No: 2)
28. Are you currently undergoing medical treatment (other than dental or psychiatric treatment)? (Yes: 1; No: 2)
29. Are you currently undergoing psychiatric treatment? (Yes: 1; No: 2)
30. Have you received psychiatric treatment before? (Yes: 1; No: 2)

Supplementary Information 2

Study 2 – Web Survey

To verify the reliability of the regression analyses findings from the first dataset (herein, Study 1) and examine the extent to which the observed relationships could be replicated, we collected a second dataset from individuals across various age groups nationwide (N = 1,029) and undertook the same analysis.

Methods

Participants

A second dataset was collected to further examine the extent to which the questionnaire results of based on the first dataset would replicate on a larger sample covering a wider range of age groups and localities. One thousand one hundred fifty respondents were sampled using quota sampling via a web survey administered by MyVoice Communications, Inc. (Tokyo, Japan) in the months of September-October 2022. Quotas were assigned based on declared gender (male/female) and age group (20-29, 30-39, 40-49, 50-59, 60-69), with 115 respondents of each gender in each age group, comprising a total sample of 1150 individuals from the entire nation. Respondents had to be residing in Japan at the time of the study and be native speakers of Japanese. They accessed the survey using a browser app on their own devices. The survey company awarded points to respondents which could later be exchanged for money.

To identify inattentive respondents, we included an instructed response item [1] in one of the scales collected during the survey. Instructed response items are easy to implement and do not alter response behavior [2, 3]. One hundred twenty-one respondents (10.5%) failed to correctly answer the instructed response item and were excluded from the analysis. The final sample consisted of 1029 respondents (510 identified as male, 519 as female), with mean age of 45.8 years old (SD = 13.8, range = 20-69). The sample covered all 47 Japanese prefectures but predominantly consisted of residents of the Kanto region (41% of the respondents). Table A summarizes the demographic characteristics of Study 2 respondents vis-à-vis the data from Study 1.

Procedure and Materials

At the start of the survey, before respondents could gain access to the questionnaire items, an initial screen was displayed with a brief description of the survey contents together with instructions. Respondents were told that all data would be anonymized before analyses and would only be used for basic research purposes. They were also told that by proceeding with the survey, they would be providing consent to participate in the data collection; if they did not wish to do so, they were told to close the browser at that point. Screening was performed in the first section of the survey; respondents were asked to declare their gender (male/female/other), their age (those whose age was outside of the range of interest, i.e., 20-69 years old, were informed about the eligibility criteria and the survey was terminated), the prefecture of residence (one of the 47 Japanese prefectures/other; those who answered 'other' were informed about the eligibility criteria and the survey was terminated), their marital status (single-divorced-widowed/married), their occupation (company employee/self-employed/highly skilled professional (e.g., MD, lawyer, etc.)/public employee/student/full-time homemaker/unemployed-retired/other), and whether they were native speakers of Japanese (yes/no; those who answered 'no' were informed about the eligibility criteria and the survey was terminated). Those who fulfilled the eligibility criteria responded to the rest of the items in the survey. After answering all the items, respondents were asked about family composition (Have children? yes/no), total household income, and personal income.

Measures

To reduce response load, the shorter 18-item version of the PWBS was employed in the web survey. Cronbach's alphas of each of the dimensions were visibly smaller compared to the values obtained in Study 1: .432 (autonomy), .467 (environmental mastery), .635 (personal growth), .542 (positive relations with others), .120 (purpose in life), and .740 (self-acceptance). Of note, the lower Cronbach's alphas obtained with the 18-item PWBS are not uncommon [4] and are a result of both the smaller number of items and a deliberate choice made by the authors of the scale [5], who reported modest

Cronbach's alpha values in their original study, ranging from .33 (Purpose in Life) to .56 (Positive Relations With Others).[1]

The 4 filler items of the LOT-R were also excluded to reduce response load; Cronbach's alpha for the 3 positive items, 3 negative items and all 6 items combined were .718, .615, and .688, respectively. The remaining scales were identical to those used in Study 1: SWLS (alpha =.908), GQ-6 (alpha =.828), SE (alpha =.893) and P-Scale (alpha =.917). Within a scale, items were presented in a random order for each respondent. Respondents also declared their current levels of happiness and stress (using the same 0-10 scale). The descriptive statistics are summarized in Table B (under Study 2).

Results

Multivariate regression analysis was performed with SWLS and the 6 PWBS dimensions as outcome variables and gratitude (GQ-6), self-esteem (SE), optimism (LOT-R), current stress, and gender (female = 0, male = 1) as predictor variables while additionally controlling for age and income (no personal income = 1, 0-200,000 yen = 2, 200,000-400,000 yen = 3, ..., 800,000 yen-1,000,000 = 6, >1000,000 yen = 7). Results are summarized in Table C. Unstandardized regression coefficients (B) indicate the sign of the relationship; effect sizes were computed in terms of partial eta squared values (η_p^2).

Gratitude was found to predict personal growth (B = 0.209, η_p^2 =.169), positive relations with others (B = 0.163, η_p^2 =.101), and self-acceptance (B = 0.150, η_p^2 =.119). To a lesser degree, gratitude

[1] The authors justified the use of the 18-item PWBS, as well as the selection of the items in each subscale as follows: *"The alpha coefficient is a conservative estimate of internal reliability for most (congeneric indicators) scales (...). The modest alpha coefficients likely reflect the small number of indicators per scale and the fact that items were chosen to represent the conceptual breadth within each construct (...) rather than to maximize internal consistency."*

was also a predictor of environmental mastery (B = 0. 055, η_p^2 =.019), purpose in life (B = 0.109, η_p^2 =.052), and SWLS (B = 0.205, η_p^2 =.050). Self-esteem was a predictor of environmental mastery (B = 0.221, η_p^2 =.164) and self-acceptance (B = 0.278, η_p^2 =.227). To a lesser degree, self-esteem was also a predictor of autonomy (B = 0.119, η_p^2 =.043), personal growth (B = 0.149, η_p^2 =.062), positive relations with others (B = 0.107, η_p^2 =.029), and SWLS (B = 0.332, η_p^2 =.081) and, to a much lesser degree, purpose in life (B = 0.048, η_p^2 =.007). Compared to gratitude and self-esteem, optimism was not a major predictor of any of the outcome variables, although it significantly explained the variance of personal growth (B = 0.080, η_p^2 =.005), positive relations with others (B = 0.140, η_p^2 =.014), self-acceptance (B = 0.171, η_p^2 =.030), and SWLS (B = 0.373, η_p^2 =.030). Optimism was also a predictor of purpose in life (B = -0.150, η_p^2 =.018), although surprisingly, the association was negative. Current level of stress was a predictor of environmental mastery (B = -0.156, η_p^2 =.025) and self-acceptance (B = -0.163, η_p^2 =.026), although for both variables, self-esteem was the most dominant predictor. Remarkably, stress was a major negative predictor of SWLS (B = -0.776, η_p^2 =.114). A minimal effect of gender was observed in SWLS (B = -0.795, η_p^2 =.005) and autonomy (B = 0.472, η_p^2 =.008), where male respondents suffered a decrease in SWLS and female respondents enjoyed increases in autonomy compared to the other gender.

Table A: Demographic characteristics of participants in the first dataset (Study 1) and Study 2. All participants were native Japanese speakers residing in Japan at the time of the study.

		Study 1			Study 2	
Participant *N*		71			1,029	
Gender						
	Female	30	41%		519	50%
	Male	41	58%		510	50%
	Other	-	-		0	0%
Age						
	20 to 29	69	97%		184	18%
	30 to 39	2	3%		199	19%
	40 to 49	-	-		211	20%
	50 to 59	-	-		212	21%
	60 to 69	-	-		223	22%
Currently a college/graduate student?						
	Yes	64	90%		23	2%
	No	7	10%		1,006	98%
Personal income						
	0-100,000 yen	57	80%	No income	149	14%
	100,001-200,000 yen	12	17%	0-200,000 yen	322	31%
	200,001-300,000 yen	2	3%	200,000-400,000 yen	248	24%
	300,001-400,000 yen	0	0%	400,000-600,000 yen	164	16%
	400,001-500,000 yen	0	0%	600,000-800,000 yen	68	7%
	>500,000 yen	0	0%	800,000-1,000,000 yen	33	3%
				>1,000,000 yen	45	4%
Married						
	Yes	-	-		473	46%
	No	-	-		556	54%

Table B: Descriptive statistics of the psychological scales. Range refers to the sample range. The shorter version (18 items) of the Psychological Well-Being Scale was employed in Study 2.

		Study 1 (*N* = 71)			Study 2 (*N* = 1,029)		
		M	*SD*	Range	*M*	*SD*	Range
NEO-FFI	1 Neuroticism	27.87	9.18	[3– 47]	-	-	-
	2 Extraversion	25.18	6.80	[11-40]	-	-	-
	3 Openness	30.82	6.56	[20-46]	-	-	-
	4 Agreeableness	30.52	6.05	[15-47]	-	-	-
	5 Conscientiousness	26.38	7.38	[11-41]	-	-	-
	6 Positivity Scale (P-Scale)	25.63	6.18	[9-40]	22.86	6.68	[8-40]
	7 Self-Esteem (SE)	26.07	5.63	[13-40]	24.95	5.79	[10-40]
	8 Satisfaction With Life Scale (SWLS)	20.28	5.78	[5-34]	18.32	6.66	[5-35]
	9 Revised Life Orientation Test (LOT-R)	15.14	2.74	[8-21]	14.50	3.00	[6-24]
PWBS	10 Autonomy	28.86	6.38	[14-49]	13.07	2.47	[3-21]
	11 Environmental Mastery	29.21	6.69	[16-47]	12.53	2.68	[3-21]
	12 Personal Growth	37.31	5.75	[23-49]	13.63	3.04	[3-21]
	13 Positive Relationships with Others	34.46	7.28	[13-46]	12.20	3.07	[3-21]
	14 Purpose in Life	31.46	5.92	[18-47]	13.46	2.50	[3-21]
	15 Self-Acceptance	30.56	7.53	[13-49]	12.67	3.45	[3-21]
	16 Gratitude Questionnaire (GQ-6)	31.77	5.61	[18-42]	27.38	5.92	[6-42]
	17 Subjective Happiness Scale (SHS)	4.69	1.25	[1-7]	-	-	-
	18 CES-D	14.80	9.83	[0-40]	-	-	-

Table C: Multivariate regression results based on data from Study 2 (N=1,029). Outcome variables are displayed in the columns, and predictor variables are displayed in the rows. Statistically significant results ($p < .05$) are highlighted in boldface - unstandardized coefficients (B), and respective effect sizes in terms of partial eta squared (η_p^2); 95% confidence intervals are in square brackets. Goodness-of-fit is indicated by the adjusted R^2.

		PWBS						SWLS
		Autonomy	Env. Mastery	Pers. Growth	Pos. Rel. Others	Purpose in Life	Self-Accept.	
	adj R^2	.113	.408	.370	.321	.076	.621	.519
Gender (Fem.=0, Male=1)	η_p^2	**.008**	.000	.001	.000	.000	.000	**.005**
	B	**.472** [.141, .804]	-.031 [-.324, .263]	.154 [-.189, .497]	-.118 [-.479, .242]	.120 [-.222, .463]	-.027 [-.329, .275]	**-.795** [-1.452, -.139]
Current stress	η_p^2	.000	**.025**	.001	.003	.003	**.026**	**.114**
	B	-.023 [-.090, .044]	**-.156** [-.215, -.096]	.042 [-.028, .111]	-.066 [-.139, .007]	.058 [-.012, .127]	**-.163** [-.224, -.102]	**-.776** [-.909, -.643]
GQ-6	η_p^2	.001	**.019**	**.169**	**.101**	**.052**	**.119**	**.050**
	B	.014 [-.014, .041]	**.055** [.031, .079]	**.209** [.181, .238]	**.163** [.134, .193]	**.109** [.080, .137]	**.150** [.125, .175]	**.205** [.150, .259]
SE	η_p^2	**.043**	**.164**	**.062**	**.029**	**.007**	**.227**	**.081**
	B	**.119** [.084, .154]	**.221** [.190, .251]	**.149** [.114, .185]	**.107** [.069, .144]	**.048** [.013, .084]	**.278** [.247, .310]	**.332** [.264, .401]
LOT-R	η_p^2	.000	.000	**.005**	**.014**	**.018**	**.030**	**.030**
	B	.023 [-.042, .089]	.003 [-.055, .061]	**.080** [.013, .148]	**.140** [.069, .211]	**-.150** [-.218, -.083]	**.171** [.112, .231]	**.373** [.244, .503]

References

1. Meade, A.W. and S.B. Craig, Identifying careless responses in survey data. Psychol Methods, 2012. 17(3): p. 437-55.
2. Gummer, T., J. Roßmann, and H. Silber, Using Instructed Response Items as Attention Checks in Web Surveys: Properties and Implementation. Sociological Methods & Research, 2021. 50(1): p. 238-264.
3. Kung, F.Y.H., N. Kwok, and D.J. Brown, Are Attention Check Questions a Threat to Scale Validity? Applied Psychology, 2018. 67(2): p. 264-283.
4. Paradise, A.W. and M.H. Kernis, Self-esteem and psychological well-being: Implications of fragile self-esteem. Journal of Social and Clinical Psychology, 2002. 21(4): p. 345-361.
5. Ryff, C.D. and C.L. Keyes, The structure of psychological well-being revisited. J Pers Soc Psychol, 1995. 69(4): p. 719-27.
